# Network Bursting Dynamics in Excitatory Cortical Neuron Cultures Results from the Combination of Different Adaptive Mechanism

**DOI:** 10.1371/journal.pone.0075824

**Published:** 2013-10-11

**Authors:** Timothée Masquelier, Gustavo Deco

**Affiliations:** 1 Unit for Brain and Cognition, Department of Information and Communication Technologies, Universitat Pompeu Fabra, Barcelona, Spain; 2 Laboratory of Neurobiology of Adaptive Processes (UMR 7102), Centre National de la Recherche Scientifique and University Pierre and Marie Curie, Paris, France; 3 Institució Catalana de la Recerca i Estudis Avançats, Universitat Pompeu Fabra, Barcelona, Spain; University of Antwerp, Belgium

## Abstract

In the brain, synchronization among cells of an assembly is a common phenomenon, and thought to be functionally relevant. Here we used an *in vitro* experimental model of cell assemblies, cortical cultures, combined with numerical simulations of a spiking neural network (SNN) to investigate how and why spontaneous synchronization occurs. In order to deal with excitation only, we pharmacologically blocked GABA_A_ergic transmission using bicuculline. Synchronous events in cortical cultures tend to involve almost every cell and to display relatively constant durations. We have thus named these “network spikes” (NS). The inter-NS-intervals (INSIs) proved to be a more interesting phenomenon. In most cortical cultures NSs typically come in series or bursts (“bursts of NSs”, BNS), with short (∼1 s) INSIs and separated by long silent intervals (tens of s), which leads to bimodal INSI distributions. This suggests that a facilitating mechanism is at work, presumably short-term synaptic facilitation, as well as two fatigue mechanisms: one with a short timescale, presumably short-term synaptic depression, and another one with a longer timescale, presumably cellular adaptation. We thus incorporated these three mechanisms into the SNN, which, indeed, produced realistic BNSs. Next, we systematically varied the recurrent excitation for various adaptation timescales. Strong excitability led to frequent, quasi-periodic BNSs (CV∼0), and weak excitability led to rare BNSs, approaching a Poisson process (CV∼1). Experimental cultures appear to operate within an intermediate weakly-synchronized regime (CV∼0.5), with an adaptation timescale in the 2–8 s range, and well described by a Poisson-with-refractory-period model. Taken together, our results demonstrate that the INSI statistics are indeed informative: they allowed us to infer the mechanisms at work, and many parameters that we cannot access experimentally.

## Introduction

It is well known that dissociated cultured neuronal networks display spontaneous activity. This activity is not steady but shows instead brief periods (0.1–0.2 s) during which most of the neurons burst – a phenomenon called “network spikes” (NS) – separated by almost silent intervals lasting several seconds [1]–[11]. Understanding how and why such synchronization occurs is crucial as synchronization is assumed to play major functional roles *in vivo*.

NS' time courses have been well characterized. For example, it has been shown that typical NS' rise time is shorter when GABA_A_ receptors are blocked [4]. Conversely, decay time is shorter when blocking NMDA receptors [11]. Typical time courses also suggest the presence of “pacemaker” neurons and adaptive synapses [9]. In comparison, the laws governing the inter-NS-intervals (INSIs) are much less understood. Certain authors have suggested that the experimentally-observed irregular NSs imply heterogeneities in either neuron properties [12] or synaptic strengths [10]. Other authors have focused on INSI distribution-tails, which has led to controversial results with evidence for both scale-free distributed INSIs [1] and narrowly-distributed INSIs [5]. However, all authors agree that some fatigue mechanism(s) must be at work to “quench” NS and to enforce a period during which subsequent NSs are much less likely, if not impossible, to occur. Yet the nature and timescales of these mechanisms are still under debate. In the vast majority of simulation studies a single fatigue mechanism was used: either cellular adaptation [2], [12], or short-term synaptic depression (STD) [6], [9], [10], [13]. Importantly, these two mechanisms are qualitatively different: adaptation completely prevents subsequent NSs, while STD only decreases their p [14] bility [14].

As we will show in this paper, it seems that realistic INSI distributions can only be obtained by using both fatigue mechanisms; adaptation having a much longer timescale than STD. Furthermore, the fact that NSs typically come in series suggests that some facilitating mechanism must be at work, most likely short-term synaptic facilitation (STF), with a timescale that must be longer than STD's, but shorter than adaptation's. We thus simulated a spiking neural network (SNN) with these three mechanisms, which, to our knowledge, had not been done before. With this we were indeed able to generate realistic NSs and BNSs. In short, we conclude that STD is responsible for quenching NSs, STF for promoting BNSs, and adaptation for interrupting BNSs and for enforcing long inter-BNS-intervals (IBNSIs).

In addition, we systematically varied the SNN excitability for several adaptation timescales. When strong, BNSs are produced almost periodically (CV∼0). When weak, BNS generation approaches a Poisson process (CV∼1). Experimental values suggest an intermediate semi-regular regime (CV∼0.5) with an adaptation timescale in the 2–8 s range. Furthermore, BNS generation, in both experiments and simulations, is reasonably well described by a Poisson-with-refractory-period model, in agreement with previous results [2]. The refractory period lasts about four times as long as the adaptation timescale.

INSIs are thus indeed informative: they allow for both the identification of the mechanisms at work and for the inference of a number of variables which we are unable to access experimentally.

## Materials and Methods

### Cell preparation

**Figure 1:**
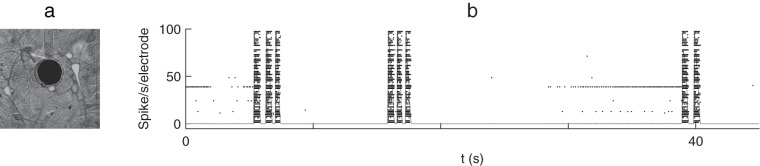
Spontaneous activity in neuronal cultures. (a) Cortical network on substrate-embedded multielectrode array. The dark circle is a 30-µm-diameter electrode. Figure is modified from ref. [4]. (b) Raster plot showing the spikes recorded at each of the 60 electrodes (black dots) as a function of time. The gray line shows the mean firing rate over 50 ms-time bins. NSs are all-or-none events, and thus easy to detect.

Cortical neurons were obtained from newborn rats (Sprague-Dawley) within 24h after birth using mechanical and enzymatic procedures described in earlier studies [15]. Rats were killed by CO_2_ inhalation according to protocols approved by the National Institutes of Health. The protocol was approved by the Inspection Committee on the Constitution of the Animal Experimentation at the Technion, approval number IL-099-08-10. The neurons were pre-treated by coating with PEI and then plated onto substrate-integrated multi-electrode arrays, and allowed to develop functionally and structurally mature networks over a time period of 2–3 wk. The number of neurons in a typical network is of the order of 1,300,000, covering an area of 380 mm^2^. The preparations were bathed in MEM (Sigma-Aldrich), and supplemented with heat-inactivated horse serum (5%), glutamine (0.5 mM), glucose (20 mM), and gentamycin (10 µg/ml). They were then maintained at 37°C, 5% CO_2_-95% air in an incubator and during the recording phases. An array of 60 Ti/Au/TiN extracellular electrodes, 30 µm in diameter and spaced 500 µm from each other [MultiChannelSystems (MCS), Reutlingen, Germany], was used (see Fig. 1a). The insulation layer (silicon nitride) was pretreated with polyethyleneimine (Sigma; 0.01% in 0.1 M borate buffer solution).

### Electrophysiological recordings

Multi-unit activity (MUA) was recorded using a commercial amplifier (MEA-1060-inv-BC; MCS, Reutlingen, Germany) with frequency limits of 150–3,000 Hz and a gain of X1,024. Data were digitized using a data acquisition board (PD2-MF-64–3M/12H; UEI, Walpole, MA). Each channel was sampled at a frequency of 16 k samples/s. Data processing was performed using a Simulink- (The Mathworks, Natick, MA, USA) based xPC target application (see ref. [16] for details). We added 6 µM bicuculline–methiodide to the bathing solution, and given that the dissociation constant is around 5 µM [17] this was assumed to block most of the inhibitory transmission.We used a total of seven recordings, each one corresponding to a different culture: one 2h-recording, four 1h-recordings, two 1/2h- recordings. Ages ranged from 14 to 35 days in vitro (DIV). Spike sorting was not attempted.

### Neuron model

The network consists in 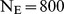 excitatory neurons. Connectivity is full. We used conductance-based leaky integrate and fire (LIF) neurons. Their membrane potential 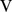 obeys the following Langevin equation:
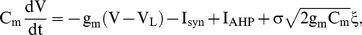
(1)Where 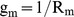 is the membrane leak conductance, 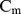 its capacitance, 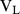 is the resting potential, 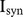 is the synaptic current (described in the next section), 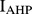 is the After-HyperPolarization adapting current (described in the Adaptation section), 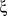 is a Gaussian white noise (with 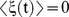 and 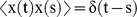), and σ is the standard deviation of the resulting noise in the membrane potential. The membrane time constant is defined by 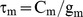. When the membrane potential reaches the threshold 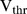 the neuron generates a spike, which is then transmitted to other neurons. Next, the membrane potential is instantaneously reset to 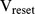 and is maintained there for a refractory time 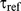, during which the neuron is unable to produce further spikes (see Table 1 for parameter values).

**Table 1:** Neuronal and synaptic parameters.

Excitatory Neurons		Synapses	
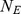	800 neurons	V_E_	0 mV
C_m_	0.5 nF	V_I_	−70 mV
g_m_	25 nS	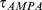	2 ms
V_L_	−70 mV	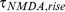	2 ms
V_thr_	−50 mV	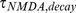	100 ms
V_reset_	−55 mV	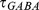	10 ms
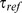	2 ms	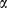	0.5 kHz
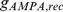	0.104 nS	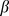	0.062
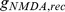	0.327 nS	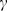	0.28
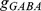	1.250 nS		

### Synapse model

**Figure 2:**
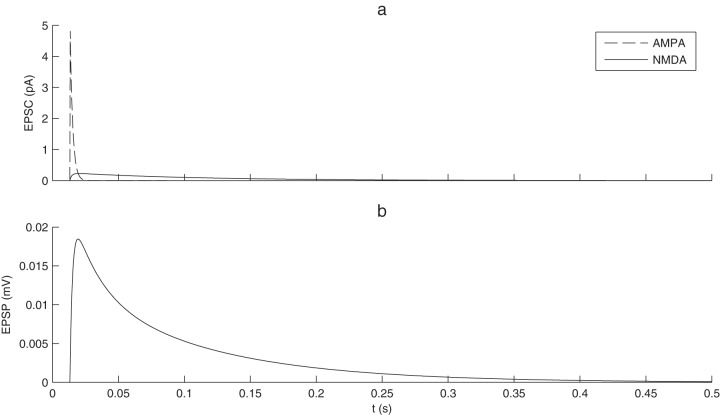
Excitatory postsynaptic currents and potentials. (a) NMDA and AMPA current dynamics after the arrival of one presynaptic spike. NMDA timescales are much longer. (b) Resulting EPSP.

Spikes arriving at a given neural synapse induce post-synaptic excitatory potentials (EPSP), essentially given by a low-pass filtering formed through the synaptic receptors. In our case, the total synaptic current is given by the sum of glutamatergic AMPA (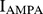) and NMDA (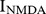) recurrent excitatory currents:
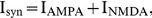
(2)where:



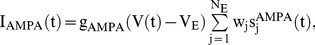
(3)

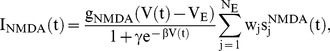
(4)Here 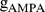 and 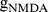 are the synaptic conductances, and 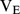 the excitatory reversal potentials. The dimensionless parameters 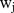 of the connections are the synaptic weights (subject to short-term plasticity, see Eq. 11). The NMDA currents are voltage-dependent and they are modulated by intracellular magnesium concentration γ. The gating variables 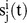 are the fractions of open channels of neurons, and are given by:



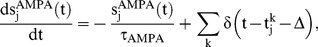
(5)

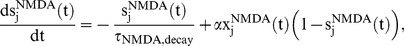
(6)

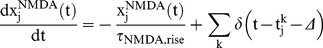
(7)Here 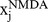 is an auxiliary gating variable for NMDA, and α is a multiplicative constant. The sums over the index 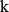 represent all the spikes emitted by the presynaptic neuron j (at times 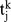). In Equations (5-7), 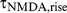 and 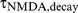 are the rise and decay times for the NMDA synapses, and 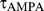 the decay times for AMPA synapses. The AMPA synapse rise time is neglected because it is very short (< 1 ms). 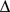 is a homogeneous conduction delay. The values of the constant parameters and default values of the free parameters used in the simulations are displayed in Table 1. Figure 2 illustrates the different time-courses of NMDA and AMPA synaptic currents and the resulting EPSP.

### Adaptation model

A spike-frequency adapting mechanism is taken into account. It is implemented in the network by including an additional leakage after-hyperpolarization current 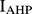 into the dynamical equation of the membrane potential of each neuron, given by the following equation:
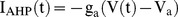
(8)where 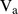 is the reversal potential and 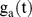 the effective additional leak conductance.


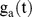
 is initially set to 0. Between spikes, it is modeled as a leaky integrator with a decay time constant 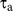:
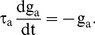
(9)


If 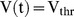, a spike is emitted and 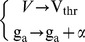
(10)


This adapting current may correspond to slow calcium- and sodium-activated potassium currents, but also to other fatigue mechanisms. 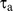 is the apparent recovery timescale of all these combined mechanisms, which, as we will see, may vary from one culture to another.

### Short-term plasticity model

All synapses are modulated by short-term plasticity (STP). The phenomenological model proposed in ref. [18] was used. It is based on the concept of the utilization of synaptic efficacy u, of which only a fraction x is available:
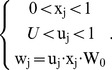
(11)


They obey a differential equation:
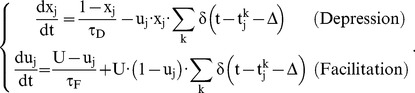
(12)


In other words, for each presynaptic spike:


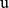
 is increased by 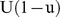



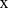
 is multiplied by 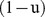.

Between presynaptic spikes:


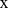
 relaxes towards 1 with time constant 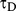



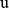
 relaxes towards 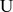 with time constant 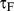


### Numerical parameters

Table 1 gathers the neuronal parameters taken from ref. [19].

An additional parameter 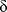 allows us to modify the ratio between AMPA and NMDA currents [20]:
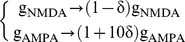



The factor 10 results from the fact that near the firing threshold the ratio of NMDA to AMPA components becomes 10 to 1 in terms of charge entry [19]. We used 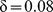, as in ref. [21].

For the Gaussian white noise, we used 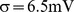.

For conduction delays (homogeneous), we used 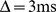 (which is in the biological range, see ref. [22]).

For STP, we used 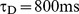 (in the biological range, see [9], [13], [14], [18], [23]), 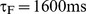, and 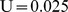 (both in the biological range, see ref. [18]). As we will see in the Result section, the mechanism that we propose for BNS requires that 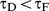, as found in ref. [18]. We note, however, that other groups have reported 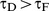 (see ref.[24] for a review).

For adaptation, we used (unless said otherwise): 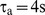, 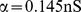, 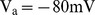.

### Simulations

We developed custom python code for the Brian clock-based simulator [25]. The differential equations were integrated numerically (Euler method) using a 25µs-time step. The code has been made available on ModelDB (http://senselab.med.yale.edu/modeldb/ShowModel.asp?model=150437).

### Detecting NS

In both the cultures and the model, NSs have an all-or-none nature and are thus easy to detect. Therefore, we just counted the spikes recorded at all electrodes in 50ms-time bins and used a threshold equal to ¼ of the maximum spike count recorded over the session. We verified that the results were not very sensitive to this threshold, nor to the bin duration.

## Results

### Spontaneous NS with bimodal INSI distributions

In all our cultures, neurons are spontaneously active (Fig. 1b), and spontaneously synchronize every 1–50 s. These synchronous events typically involve the whole network [4], hence the denomination “network spikes”. While the firing rates during such NSs can reach 100 spikes/electrode/s or more, it is typically of ∼1spike/electrode/s between NSs or less (Fig 1b, gray line). NSs are thus easy to detect, for example using a simple rate threshold (see Materials and Methods). The results are not very sensitive to the threshold nor to the time bin size.

**Figure 3:**
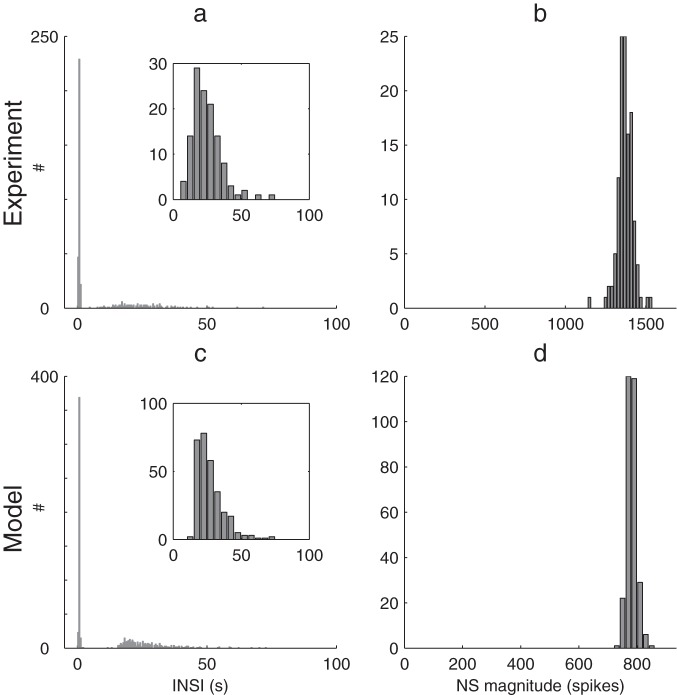
INSI and NS magnitude histograms. (a) Experimental INSI distribution in one representative culture, using 500 ms time bins. The distribution is bimodal, with either very short INSIs (<1.5 s) or much longer ones (>6 s).The inset zooms on the long INSIs (>6 s) and uses a larger time bin (5 s). Notice the (somewhat) long tail: the distribution is positively skewed. (b) Conversely, the experimental NS magnitude distribution is unimodal and quite narrow (only the first NS of each BNS is taken into account). (c and d) Same histograms for the model, with 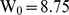 and 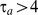 s (Note that we fitted the NS timescales, but not their magnitudes. Indeed, we do not know how many neurons each electrode picks on average; therefore we cannot estimate the true average number of spikes per second per neuron).

In most cultures, NSs are not “isolated”, but come in series (“burst of NSs”, BNS) with short INSIs (∼1 s or less), while the intervals between BNSs are substantially longer (tens of s), leading to bimodal INSI distributions (Fig. 3a). Importantly, these BNSs are also commonly seen without blocking the GABA_A_ receptors [5]. The inset zooms in on the long INSIs (>6 s). Notably, their distribution is slightly positively skewed, with a somewhat long tail (we will come back to this point in the Working point identification section).

Conversely, the NS magnitudes, expressed here in total number of spikes emitted, appear to be normally distributed (Fig. 3b), with a small standard deviation (lower than 4% of the mean). Note, however, that we only included the first NS of each series; subsequent ones tend to be weaker due to fatigue mechanisms. Durations and maximum firing rates are also narrowly distributed (data not shown). This confirms the all-or-none nature of NSs previously reported in similar preparations, especially when using bicuculline [4]. Magnitude statistics are thus not very informative here.

Our goal was now to come up with a minimal spiking neural network (SNN) model. It had to be as simple as possible, and yet able to produce all-or-none NSs with bimodal INSI distributions (Fig. 3c and d). What were the key “ingredients” needed? As mentioned above, GABA_A_ receptors were blocked in the experimental recordings. Thus, inhibitory neurons were not expected to impact on the dynamics and for the sake of simplicity we thus ignored them in our model.

### Implications for the mechanisms at work

To trigger an NS, two things are needed: random fluctuations and positive feedback [12]. In our model, the random fluctuations are caused by the Gaussian white noise (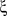 in Eq. (1)). The recurrent excitatory connections, whose strength can be adjusted (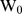 in Eq. (11)), provide the positive feedback, causing an exponential recruitment of cell activity in the early phase of each NS [12], as observed experimentally [4].

Next, what are the mechanisms responsible for quenching the NS? The candidate restoring forces are: the recruitment of the inhibitory network (which can be discarded when blocking GABA_A_ receptors like here), cellular refractoriness and adaptation, and short-term synaptic depression (STD). The long (∼20–30 s), semi-regular (CV∼0.5) inter-BNS-intervals (IBNSI, Fig. 3a inset) imply that at least one of these mechanisms has a long timescale (∼2–8 s, see Working point identification section). This is most probably adaptation or STD, since cellular refractoriness is typically in the millisecond range.

Besides, as said above, INSI distributions are often bimodal. In other words, the probability of getting an NS is higher if there was another one in a recent past (∼1 s), even if activity has returned to baseline. This suggests that a facilitating mechanism is at work, most probably short-term synaptic facilitation (STF). Alternatively, this could be due to waves of activity reverberating along the network border, where connectivity is denser, and returning to the point where the NS started with sufficient delay to find neurons ready to fire again and ignite another NS [23]. This possibility, demonstrated in large-scale simulations (10,000–50,000 neurons) [23], is not considered further in this paper. Hence we assume that STF is at work, with a time constant 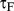 in the 1–3 s range (which is in broad agreement with the experimental estimation of 1797±1247 ms [18]), and that there is at least one fatigue mechanism with a long timescale (∼2–8 s). This left us with four possible scenarios (Table 2): the long fatigue timescale could correspond to STD, or to cellular adaptation; and there could be two or just one fatigue mechanism. To rule these four scenarios in or out we performed exhaustive searches on the numerical parameters (Table 2).

**Table 2:** Ruling in and ruling out fatigue mechanisms.

The long fatigue timescale is:1 or 2 fatigue mechanisms:	Adaptation	STD
Adaptation XOR STD	Case A (adaptation only): no BNS	Case B (STD only): no BNS, at least if 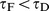
Adaptation AND STD	**Case C: BNS possible**	Case D: no BNS, at least if 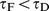

Using strong adaptation (high α in Eq. 10) we observed that it is possible to quench an NS without STD (case A in Table 2). But each NS is then followed by a period in which the network is completely silent (data not shown). Thus facilitation, which modulates input spike effect, did not help in triggering subsequent NSs, and the network only produced isolated NSs. In other words, we confirmed the results of ref. [14]: adaptation enforces a hard refractory period, which prevents BNSs, while STD only decreases NS probability, which can be compensated by STF. Case A was thus ruled out.

Using only STD with a long timescale 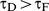 (case B), again we were only able to produce isolated NSs. Indeed, if 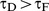, then the net effect of short-term plasticity (STP) shortly after an NS is depressing and does not promote subsequent NSs. Obviously, adding adaptation here (case D) did not help us in getting BNSs. We verified that it is possible to produce BNSs using 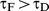 ∼2–8 s. However, not only were these time constants not very realistic, but the intra-BNS INSIs were several seconds long, in contradiction with experimental observation (1 s or less, see Fig. 3a). Accordingly, both cases B and D were largely ruled out.

**Figure 4:**
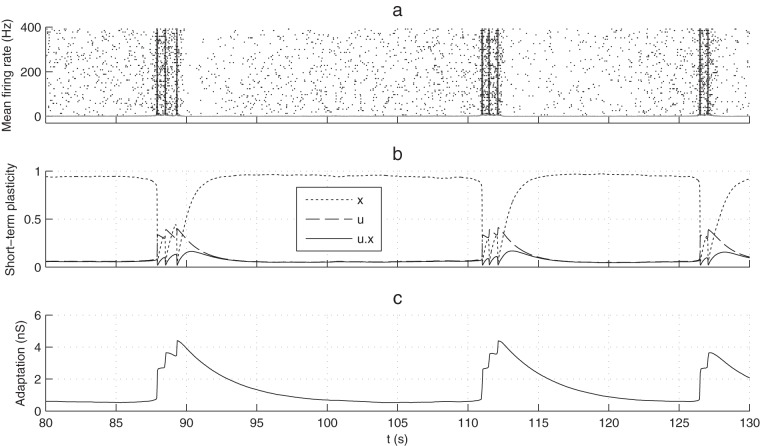
Model neurodynamics. (a) Raster plot showing the spikes of 60 neurons (black dots) as a function of time. The gray line shows the mean population firing rate (b) STP variables (population-averaged) as a function of time. Notice that, since 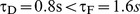, depression X recovers faster than facilitation u after an NS. Thus the net STP modulation u.X shortly after an NS is facilitating, promoting subsequent NSs. (c) Adaptation leak conductance g_a_ (population-averaged) as a function of time. It accumulates after each NS. When too strong, the NS series is interrupted, and followed by a long silent interval.

That left us with case C. STD is at work with a short timescale 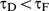 (respectively 0.8 and 1.6 s in the baseline simulation, see Fig. 4). Hence the net result of STP shortly after an NS is facilitative (Fig. 4b), promoting BNSs. Long timescale (weak) adaptation is responsible for both interrupting the series, and for enforcing long IBNSIs (Fig. 4c). However, the main restoring force quenching each NS is STD, in line with experimentation [26] and seminal modeling work [13].

Note that this reasoning would not have been possible without blocking GABA_A_ receptors, because inhibition would have provided a third restoring force, of which the timescale would be unclear [23]. But of course it seems reasonable to assume that the mechanisms we have identified, namely STP and cellular adaptation, with 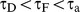, are also at work when GABA_A_ receptors are unblocked – although obviously the resulting dynamics changes.

### Fatigue timescales' separation

**Figure 5:**
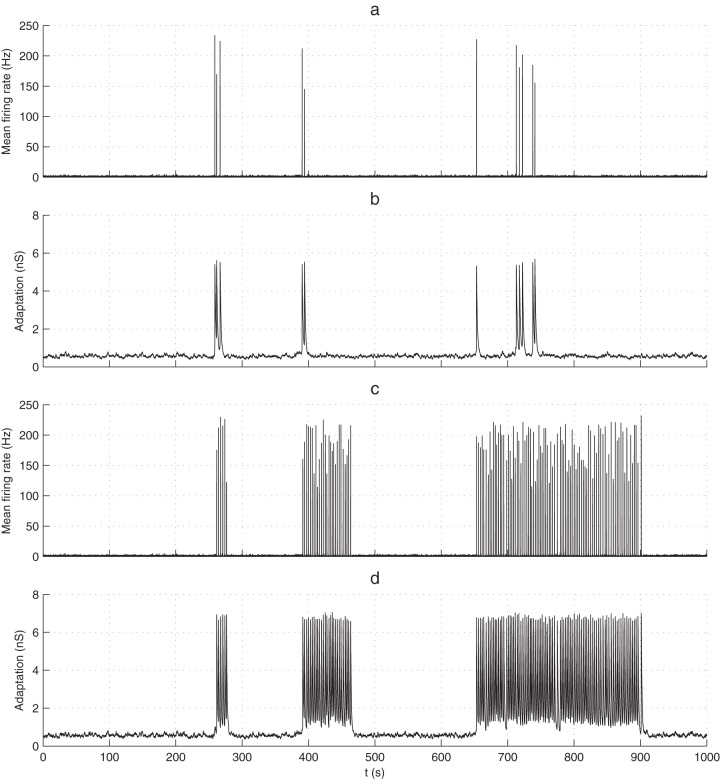
Fatigue timescales' separation. For panels (a–b) 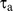 = 1.6 s, while for panels (c–d) 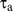 = 1.2 s. In all cases, 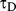 = 0.8 s, 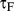 = 1.6 s, and W_0_ = 8.6 (a) Mean population firing rate as a function of time. (b) Adaptation leak conductance g_a_ (population-averaged) as a function of time. Inside a BNS, it tends to accumulate across successive NSs, until it is high enough to prevent subsequent NSs. Thus BNS termination is almost deterministic, while BNS initiation is stochastic. (c) Mean population firing rate as a function of time. (d) Adaptation leak conductance g_a_ (population-averaged) as a function of time. It cannot accumulate across successive NSs. Thus both BNS initiation and termination are stochastic. This can lead to hundreds of ms long BNSs, which is not realistic.

As we have explained above, we suggest that STP with its time constants 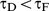 shapes short intra-BNS INSIs, while adaptation, with a longer time constant 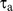, shapes the long IBNSIs. To what extent did 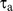 need to be greater than 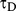 and 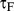 in order to obtain realistic BNSs? We kept 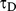 = 0.8 s and 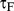 = 1.6 s, and performed an exhaustive search on 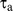, keeping 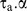 constant (to maintain the global level of adaption, see Eq. 9–10). It turned out that a qualitative change of regime occurs between 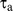 = 1.2 s and 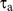 = 1.6 s (Fig. 5).

For 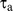 = 1.6 s (as in Fig. 4), adaptation conductance can accumulate across intra-BNS NSs until it is high enough to prevent subsequent NSs and the series terminates (Fig. 5a and b). In this regime, BNS initiation is stochastic. What happens next, however (exponential recruitment, STD-induced activity decay, STF-induced subsequent NSs, adaptation-induced series termination), is much more deterministic, yet not fully so. A similar regime has been identified in SNNs with adaptation but without STP, and thus without resulting in BNSs [27].

For 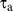 = 1.2 s, the adaptation conductance cannot accumulate across intra-BNS NSs. Thus, it will not systematically terminate the BNS. Instead, BNS termination is stochastic, which can lead to very long BNSs (Fig. 5c and d). Such long BNSs have not been observed in our cultures.

Hence, to get a clear separation of STP and adaptation timescales, a necessity to get realistic BNSs, 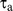 needs to be greater than 1.2 s. This is consistent with the experimental range estimated in the next section: 2–8 s.

### Working point identification

**Figure 6:**
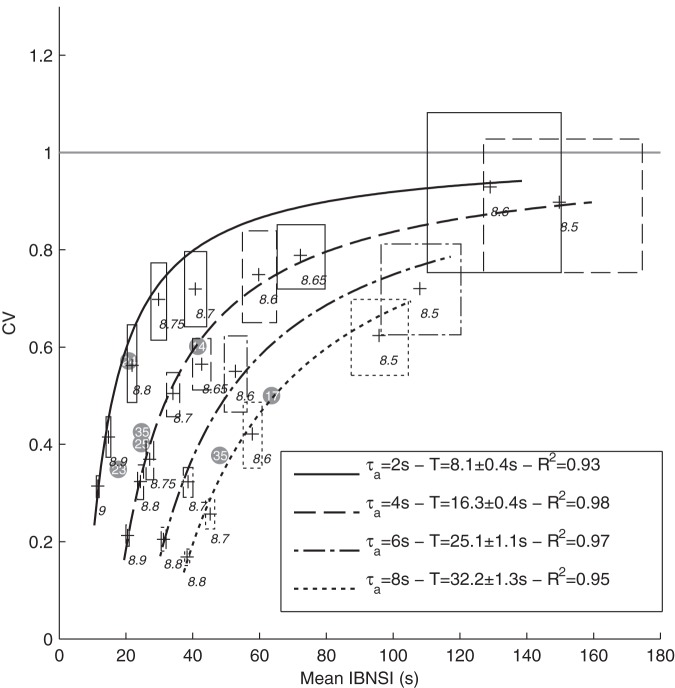
Working point. Each curve corresponds to all the possible (Mean IBNSI, CV) points that the model can reach by varying the recurrent excitatory weight W_0_ (see values in italic), keeping 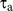 constant. Simulations stopped when 300 BNSs were recorded (with an additional 5h limit). Cases with less than 100 BNSs were discarded. The boxes represent 95% confidence intervals (estimated with bootstrapping), for both CV and mean IBNSI. We fitted a Poisson-with-refractory-period model CV  =  (mean-T)/mean for each 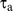 value. The legend shows estimated refractory periods T, with 95% confidence intervals, and the coefficients of determination R^2^. Gray circles represent the experimental cultures, and the numbers inside are the corresponding days in vitro (DIV), which did not correlate with the mean INSIs, or the CV. The horizontal gray line materializes the asymptotic limit CV = 1.

In the following section, we will focus on long IBNSIs (>6 s, see Fig. 3a and 3c insets). As explained above, two things are needed to trigger an NS: a random fluctuation of a certain magnitude, and a recurrent excitation that provides positive feedback. We systematically varied excitability in the model (i.e. the recurrent excitatory weight 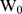 in Eq. 11), keeping the noise parameter 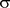 constant. As expected, the BNS frequency (respectively the mean IBNSI) increased (resp. decreased) with 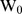 (Fig. 6), because smaller fluctuations become sufficient to trigger NSs.

**Figure 7:**
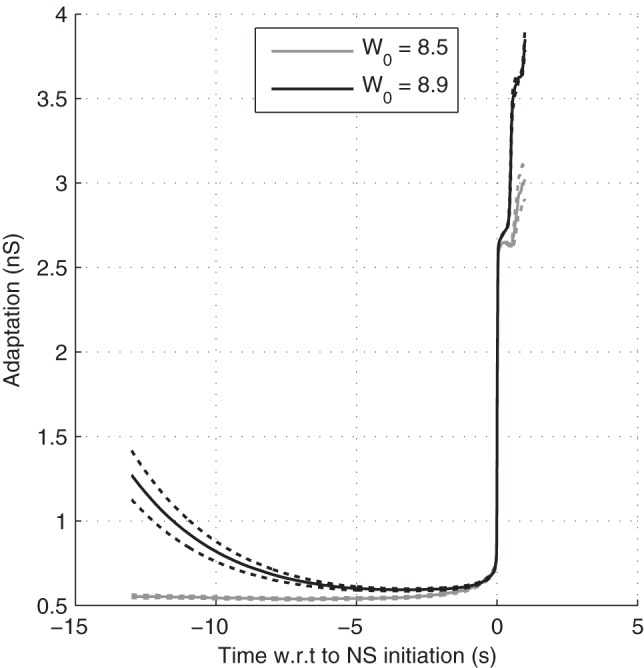
Adaptation in regular vs. irregular mode. Here we plotted the population-averaged adaptation leak conductance g_a_ preceding the BNSs, averaged across 100 BNSs, with W_0_ =  8.5 and 8.9 (and 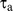 = 4 s). Dotted lines show 95% confidence interval for the mean.

In addition, the shape of the IBNSI distribution changes. In the case of large excitability as soon as the adaptation-induced refractory period is over (say, after a few 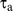
s), an NS is rapidly produced, because the required weak fluctuation appears quickly (see also Fig. 7). Consequently, the IBNSI are regular; that is, their coefficient of variation (CV) is low (Fig. 6). Conversely, with low excitability, the required strong fluctuation may take longer to appear, leading to a positively skewed IBNSI distribution.

In the limit case 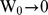, neurons are independent. They spike only due to the independent white noise they receive (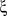 in Eq. 1), with very long mean inter spike interval (∼2 s with our parameters). At such low rates spike generation approaches a Poisson process (individual CV ∼1). Thus, spike coincidences between neurons, which cause the required fluctuations, also follow a Poisson process. As a result, the IBNSI CV approaches 1, while NS becomes extremely rare (Fig. 6). It is worth noting that even when 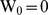, one is guaranteed to see an NS if one waits long enough, as at some point the independent neurons will fire synchronously. In practice, however, and with reasonable time bins and thresholds for NS detection, the expected waiting time would be astronomical.

In addition, we also varied the adaptation time constant 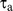 (Fig. 6), from 2 to 8 s, keeping 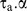 constant (to maintain the global level of adaption). This shifted the curve of all possible (CV, Mean INSI) values either up or down.

We then wanted to test how well BNS generation in the model may be described by a Poisson point process with refractory period *T* [28]. In such processes, the *CV* is linked to the mean interval *m* by 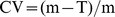. We thus fitted one model of this kind for each 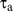 value (Fig. 6), adjusting *T*. We used the non-linear least square method and we assigned a weight to each data point that was inversely proportional to its error box area. The legend shows estimated refractory periods *T*, which turned out to be around 4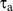. This confirms that the cause here of the refractory period is adaptation, as opposed to STD. Overall, the fittings were good (R^2^≥0.93), indicating that the Poisson process with refractory period sis a reasonable model for BNSs. This was also the conclusion of a previous simulation study [2], which did not, however, include STP, and thus had only isolated NSs. The goodness of fit also confirms that adaptation has the capacity to enforce a hard refractory period [14].

Finally, we placed our seven experimental data points, corresponding to the different cultures on the (Mean IBNSI, CV) plane (Fig. 6, gray circles). This allowed us to determine for each culture, and without ambiguity, the apparent timescale of adaptation 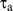 (given by the unique curve passing through the experimental point), which turned out to be in the 2–8 s range, and then the strength of recurrent excitation 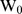 needed to reach that particular point on the curve.This strength could be mapped to the density of excitatory synapses. Remarkably, this inference was possible using the IBNSI statistics only. All cultures appeared to operate in an intermediate, weakly-synchronized regime with semi-regular BNS (CV∼0.5).

The Kolmogorov-Smirnov statistical test used in ref. [28] failed to provide evidence for power-law distributed, rather than exponentially-distributed IBNSIs, in both experimental and simulated data. This is again consistent with the Poisson point process with refractory period, which leads to interval distributions with exponentially decaying tails.

We also plotted the NS-triggered average of the adaptation leak conductance g_a_, in case of strong (respectively weak) recurrent excitation producing regular (resp. irregular) NSs (Fig. 7). As was expected in the regular case, a relaxed adaptation conductance is a necessary and sufficient condition for NS generation (black curve). In the irregular case, it was only a necessary condition. A significant fluctuation also needs to appear and this may take some time During this waiting period the adaptation conductance was typically low (gray curve).

## Discussion

Time series of network synchronization events (NSs) demonstrate complex statistics, both *in vivo* and *in vitro*. Current models fall short of explaining these statistics. Here, we have shown that the incorporation of three adaptive processes (short-term synaptic facilitation, fast and slow fatigue) is sufficient to reconstruct the experimentally- observed complex statistics. Our theoretical framework was adjusted and validated by fitting to a data set we had obtained from *in vitro* large-scale networks of excitatory cortical neurons.

INSI statistics appear to have been somewhat overlooked by many of the community. Most researchers focus on characterizing and explaining NSs' magnitudes and durations. For example, in a number of preparations, these two variables have been shown to follow power-law (scale-free) distributions [7], [8], much like the so-called “neuronal avalanches” in organotypic cultures and acute cortical slices [29]. These power-laws, which seem to hold only with inhibition [11], [29], [30] have been interpreted as a signature of self-organized criticality (but see ref. [31], [32]), which is theoretically appealing [29], [30], [33], [34].

In our experiments inhibition was blocked, and therefore NS magnitudes were narrowly distributed. However, INSI statistics turned out to be more interesting. They enabled us, firstly, to infer the mechanisms at work, and secondly to estimate their timescales. More specifically, bimodal INSI distributions suggest that both STP and adaptation are at work, with 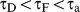. In short, STD is responsible for quenching the NS, STF for promoting BNSs, and adaptation for interrupting the BNSs and enforcing long IBNSIs. The long modes of the INSI distributions unambiguously determine variables which we cannot access experimentally, namely 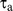 and the excitability. In turn, the variables determine the system's working point. With strong excitability, IBNSI are short and regular (low CV), while with weak excitability NS generation approaches a Poisson process with extremely long irregular IBNSIs (CV∼1). It seems that experimental cultures operate in an intermediate mode, producing semi-regular IBNSIs (CV∼0.5) with a slightly positively-skewed distribution. A Poisson-with-refractory-period model fits well with both simulated and experimental IBNSI.

Importantly, we used a simple full non-plastic connectivity (although random sparse ones gave similar results, data not shown). It is likely that some modularity is needed to obtain more graded, possibly power-law-distributed, NS magnitudes [34]. Spike timing-dependent plasticity may also help [34]. However, evidence for such power-laws in dissociated cultured neuronal networks remains rare [7], [8]. In our experiments we observed stereotyped almost all-or-none NSs, in line with previous reports [4]. Power-law-distributed sizes and durations are more established in organotypic cultures and acute cortical slices [29], [35], possibly because they have the required connectivity.

Remarkably, a full connectivity with homogeneous synaptic weights turned out to be sufficient to capture the experimentally-observed INSI statistics. Our neurons were indistinguishable, and yet we observed non-periodic NSs. This could be seen as incongruent with simulations that have shown that non-homogeneous cell properties [12] or synaptic weights [9] are required for non-periodic synchronization. We admit, however, to injecting a fair amount of independent white noise current to each neuron. This noise may be playing a similar role as non-homogeneities.

However, a full connectivity with homogeneous weights is clearly a limitation. Among other things, we cannot capture the fact that some neurons are much more active than others, and that an upcoming NS can be reliably predicted based on the activity of a few “privileged” neurons as early as 100ms before the NS peak [4].

NS in neuron cultures is a robust and well-documented phenomenon [1]–[11]. These *in vitro* preparations offer an ideal framework to combine experimental work with simulations. Experimental conditions can be much more carefully controlled than in most *in vivo* experiments. In particular, the external inputs may be fully controlled or canceled (as in our experiments), and pharmacological manipulations are also possible. However, it is worth mentioning that NS-like synchronous events have been observed *in vivo* as well: in the cortex [36]–[38], the hippocampus [39], and in LGN [40].

Synchronization is thought to play major functional roles in the brain. Individual neurons are sensitive to synchronous inputs [41], [42] and these are favored by synaptic plasticity mechanisms [43]. At the level of cell assembly, synchrony is thought to enable feature binding [44], communication through coherence [20], [45], and phase-of-firing coding [46], [47]. Understanding how and why synchronization occurs is thus of crucial importance, and as we have argued here, *in vitro* neuronal cultures might be useful as an experimental model for generic cell assembly. One must, however, keep in mind the obvious constraints on extrapolations from *in vitro* to *in vivo* conditions, in particular because the networks are no longer isolated (for review, see ref. [15], [48]).
