# Correction: Network Bursting Dynamics in Excitatory Cortical Neuron Cultures Results from the Combination of Different Adaptive Mechanism

**DOI:** 10.1371/annotation/b7f972c2-57df-44c4-9d54-9f194222d762

**Published:** 2013-11-08

**Authors:** Timothée Masquelier, Gustavo Deco

The word "mechanisms" was misspelled in the title of the article. The correct title is: Network Bursting Dynamics in Excitatory Cortical Neuron Cultures Results from the Combination of Different Adaptive Mechanisms. The correct citation is: Masquelier T, Deco G (2013) Network Bursting Dynamics in Excitatory Cortical Neuron Cultures Results from the Combination of Different Adaptive Mechanisms. PLoS ONE 8(10): e75824. doi:10.1371/journal.pone.0075824 

